# Redefining Low Lead Levels

**DOI:** 10.1289/ehp.1103489

**Published:** 2011-05

**Authors:** Ivo Iavicoli, Edward J. Calabrese

**Affiliations:** Istituto di Medicina del Lavoro, Facoltà di Medicina e Chirurgia, Università Cattolica del Sacro Cuore, Rome, Italy, E-mail: iavicoli.ivo@rm.unicatt.it; Environmental Health Sciences, School of Public Health, University of Massachusetts, Amherst, Massachusetts

In the January 2011 issue of *EHP*, Giddabasappa et al. (2011) reported that gestational lead exposure (GLE) of C57BL/6 mice produced selective nonmonotonic increases in the numbers of rods and cone bipolar cells (BCs) in the adult retina. Interestingly, this increase was characterized by an inverted U-shaped dose–response curve. Moreover, findings of this study showed that GLE increases and prolongs proliferation of retinal progenitor cells (RPCs) without decreasing apoptosis. Consequently, this phenomenon produced an adult retina with normal lamination and a selectively increased number of rods and BCs. These results should be considered to define a more adequate risk assessment at low levels of lead exposure. In fact, other published articles have indicated that lead induced a biphasic dose–response relationship (Calabrese and Baldwin 2003).

In experiments in Swiss mice using low-level lead exposures similar to and lower than those used by Giddabasappa et al. (2011), we observed an increase in the number of red blood cells, in female gestational parameters, and in Th1 cytokine levels (Iavicoli et al. 2003, 2004, 2006a, 2006b). For this reason, it would be interesting if Giddabasappa et al. could verify this increase in the number of neurons in the rod-signaling pathway at even lower blood lead levels (< 10 μg/dL). The findings of our studies were also implicated over several generations.

In any case, we agree with Giddabasappa et al. (2011) that their findings, as ours, raise complex issue for toxicologists, pediatricians, public health regulators, and risk assessors who need to incorporate the occurrence of such U-shaped dose responses in the hazard and risk assessment process. In this context, these findings could be explained by the hormesis phenomenon, which is a dose–response relationship characterized by low-dose stimulation and high-dose inhibition (Calabrese 2008, 2009).

## Editor’s note

In accordance with journal policy, Giddabasappa et al. were asked whether they wanted to respond to this letter, but they chose not to do so.
